# Response to “The opinion of French pulmonologists and palliative care physicians on non-invasive ventilation during palliative sedation at end of life: a nationwide survey’’

**DOI:** 10.1186/s12904-023-01130-7

**Published:** 2023-03-06

**Authors:** Robert Twycross, Christina Faull, David Wenzel, David Oliver

**Affiliations:** 1 Palliative Medicine, University of Oxford, Oxford, UK; 2 Palliative Care, De Montfort University, Leicester, UK; 3 Palliative Medicine & Research Lead, LOROS Hospice, Groby Road, Leicester, LE3 9QE UK; 4 Palliative Medicine, LOROS Hospice, Groby Road, Leicester, LE3 9QE UK; 5 Palliative Care, Tizard Centre, University of Kent, Canterbury, UK

**Keywords:** End of life, Non-invasive ventilation, Discontinuation of treatment, Palliative sedation

## Abstract

We have several major concerns about this article [Guastella et al 2021]. Although it states that it is about palliative sedation, it is not. Rather, it is about the French Claeys Leonetti Law about Continuous Deep Sedation (CDS) at the end of life [Loi n°2016-87].

We have several major concerns about this article (Guastella et al 2021). Although it states that it is about palliative sedation, it is not. Rather, it is about the French Claeys Leonetti Law about Continuous Deep Sedation (CDS) at the end of life (Loi n°2016-87 du 2 Février 2016 créant de nouveaux droits en faveur des malades et des personnes en fin de vie 2016). Although CDS and palliative sedation overlap, they are not synonymous: palliative sedation by definition is proportionate to need and may range from light to deep (Broeckhaert 2011). Not maintaining this distinction has led, and still leads, to considerable confusion in the literature (Twycross 2019, Kremling & Schildmann 2020). Here it is inexcusable given that the French Law is specifically about CDS (Bretonnaire & Fournier 2021).

It is the second clause of the Claeys Leonetti Law which is relevant here: CDS for the avoidance of all suffering if life-sustaining treatments are stopped. In relation to non-invasive ventilation (NIV), this means *anticipatory sedation* to prevent anxiety, breathlessness, and feelings of suffocation when withdrawn. It is probably best thought of as a form of procedural sedation (Green 2021), not as CDS. Thus, its inclusion in the Claeys Leonetti Law may hinder rather than help.

The authors refer to the European Association for Palliative Care (EAPC) recommended framework for the use of sedation in palliative care (Cherny & Radbruch 2009). However, this did not discuss sedation when withdrawing assisted ventilation because guidelines already existed. In the UK, we have the ‘Guidance for Professionals’ produced principally by the Association for Palliative Medicine (APM) (Faull & Oliver 2016). Although specifically about motor neurone disease/amyotrophic lateral sclerosis (MND/ALS), the principles and recommendations are applicable in patients supported by assisted ventilation from any cause of irreversible respiratory failure. The issue is approached from general medical ethical considerations, and not through the ‘straitjacket’ of a legal statute.

France is the only country in the world to have a Law which enshrines a patient’s right to have CDS in certain circumstances at the end of life. However, we suggest that the Law may have unintentionally induced a form of ‘tunnel vision’ in French physicians in relation to the use of sedatives at the end of life. Physicians have an over-arching commitment both to sustain life when feasible and to relieve suffering in all circumstances (‘Guerir quelquefois, soulager souvent, consoler toujours’). They cannot standby and do nothing. However, even so, they must work within the broad principle of ‘do good, minimize harm’ (beneficence, non-maleficence) (Beauchamp & Childress 2013). Thus, in the UK, clinical practice is well summed up in a legal judgement of 1958:

‘A doctor who is aiding the sick and the dying does not have to calculate in minutes or even in hours, and perhaps not in days or weeks, the effect upon a patient’s life of the medicines which he administers or else be in peril of a charge of murder. If the first purpose of medicine, the restoration of health, can no longer be achieved, there is still 2 much for a doctor to do, and he is entitled to do all that is proper and necessary to relieve pain and suffering, even if the measures he takes may incidentally shorten life’ (Devlin 1985).

We suggest that such sentiments reflect a broad international clinical consensus – *acting on the basis of necessity to relieve suffering while seeking to minimize risk to life as far as possible*. Nonetheless, even at the end of life, the intended aim of treatment should be the relief of suffering and not the patient’s death (World Health Organization 2002). A greater risk to life is acceptable in more extreme circumstances, but measures which carry less risk to life should be used in the first place. Thus, *as a last resort*, it is acceptable to render a patient unconscious because less extreme measures have failed to bring adequate relief, but there is still need for proportionality (Brockaert 2011).

It is important to note that anticipatory sedation before NIV withdrawal does not necessarily mean CDS (Faull & Wenzel 2020). Further, maintaining NIV after establishing a satisfactory level of sedation, demonstrates a failure to understand the ethics of sedation and the reason for it in this situation.

As the authors acknowledge, the binary nature of the Survey’s questions make it hard to answer meaningfully, particularly without any context except end of life. The inclusion of a reference to an article specifically about MND/ALS may indicate that this was foremost in their thinking (Bourke 2006). However, for pulmonologists, other causes of end-stage respiratory failure would quite likely spring to mind.

Nonetheless, we agree that the most important finding in the Survey is the difference in the percentage of pulmonologists and palliative care physicians who maintain NIV when starting CDS: ≈28% versus ≈12%. Regrettably, this difference is repeatedly obscured in the article by merging the two groups (≈20%). However, the demonstration that palliative care training was associated with a more positive attitude toward NIV withdrawal emphasizes the need for close collaboration between palliative care specialists and pulmonologists.

## Data Availability

Not applicable.

## Abbreviations

APM: Association for Palliative Medicine of Great Britain and Ireland
CDS: Continuous Deep Sedation
EAPC: European Association for Palliative Care
MND/ALS: Motor neurone disease/amyotrophic lateral sclerosis
NIV: Non-invasive ventilation
UK: United Kingdom of Great Britain and Northern Ireland
